# Factors influencing the outcome of paediatric cardiac surgical patients during extracorporeal circulatory support

**DOI:** 10.1186/1749-8090-2-4

**Published:** 2007-01-11

**Authors:** Sendhil K Balasubramanian, Ravindranath Tiruvoipati, Mohammed Amin, Kanakkande K Aabideen, Giles J Peek, Andrew W Sosnowski, Richard K Firmin

**Affiliations:** 1 Department of ECMO, Glenfield General Hospital, Leicester, LE3 9QQ, UK; 2 Department of Paediatric cardiology, Glenfield General Hospital, Leicester, LE3 9QQ, UK

## Abstract

**Background:**

Veno-arterial extracorporeal membrane oxygenation (ECMO) is a common modality of circulatory assist device used in children. We assessed the outcome of children who had ECMO following repair of congenital cardiac defects (CCD) and identified the risk factors associated with hospital mortality.

**Methods:**

From April 1990 to December 2003, 53 patients required ECMO following surgical correction of CCD. Retrospectively collected data was analyzed with univariate and multivariate logistic regression analysis.

**Results:**

Median age and weight of the patients were 150 days and 5.4 kgs respectively. The indications for ECMO were low cardiac output in 16, failure to wean cardiopulmonary bypass in 13, cardiac arrest in 10 and cardio-respiratory failure in 14 patients. The mean duration of ECMO was 143 hours. Weaning off from ECMO was successful in 66% and of these 83% were survival to hospital-discharge. 37.7% of patients were alive for the mean follow-up period of 75 months. On univariate analysis, arrhythmias, ECMO duration >168 hours, bleeding complications, renal replacement therapy on ECMO, arrhythmias and cardiac arrest after ECMO were associated with hospital mortality.

On multivariate analysis, abnormal neurology, bleeding complications and arrhythmias after ECMO were associated with hospital mortality. Extra and intra-thoracic cannulations were used in 79% and 21% of patients respectively and extra-thoracic cannulation had significantly less bleeding complications (p = 0.031).

**Conclusion:**

ECMO provides an effective circulatory support following surgical repair of CCD in children. Extra-thoracic cannulation is associated with less bleeding complications. Abnormal neurology, bleeding complications on ECMO and arrhythmias after ECMO are poor prognostic indicators for hospital survival.

## Background

Mechanical circulatory support plays a critical role in the management of medically refractory post cardiotomy circulatory failure. Various circulatory assist devices like Intra aortic balloon pump (IABP), Ventricular assist devices (VAD) and Venoarterial Extracorporeal membrane oxygenation (VA-ECMO) have been successfully used in postcardiotomy cardiogenic shock [1-3]. VA-ECMO is the most commonly used therapy, especially in paediatric patients, following repair of congenital cardiac defects. This is due to the advantage of provision of pulmonary as well as cardiac support by VA-ECMO and also ready availability and familiarity of ECMO circuits in pediatric intensive care units (PICU). Moreover other types of circulatory assist devices have their own limitations in pediatric use [1].

The first application of ECMO as a cardiac support following a palliative repair of congenital heart disease in a pediatric patient was done by Baffes et al in 1970 [4]. Over the past decade, the number of children successfully treated by ECMO has increased gradually[3,5-9]. Since the practice and outcome of ECMO varies widely among the different centers around the world, we studied the long-term outcome of children who had circulatory support with VA-ECMO following repair of congenital heart defects in our institution, with a view to identify the possible risk factors associated with hospital mortality. We also explored the relationship between the type of cannulation and bleeding complications during ECMO support.

## Methods

From April 1990 to December 2003, a total of fifty-three patients required VA ECMO following the surgical correction of congenital cardiac defects in our institution, Glenfield Hospital U.K. This represents 3.2% of total number of congenital heart operations performed using cardiopulmonary bypass during this period. Data was collected retrospectively from patient's medical records, operative reports, cardiac catheterization laboratory database, ECMO department and PICU database. Follow-up information was obtained from outpatient clinic and telephone enquiry with patient's general practitioner. Patient characteristic and diagnosis were shown in Table 1.

**Table 1:** Patient characteristics and diagnosis

**Variables**	**Survivors**	**Non survivors**	**Total**
Male	15	14	29
Female	14	10	24
Median Age	142 days	180 days	-
Median Weight	6 kg	5.2 kg	-
Mean duration of ECMO	94 hr	164 hr	-

**Anatomical Diagnosis**			

Tetralogy of Fallot's (TOF)	4	5	16
TOF with additional lesions^a^	2	5	
Transposition of great arteries (TGA)	5	2	10
TGA with additional lesions^b^	1	2	
Ventricular septal defects (VSD)	2	1	6
VSD with additional lesions c	2	1	
Total anomalus pulmonary venous drainage	2	2	4
Atrioventricular septal defect (AVSD)	2	2	4
Truncus arteriosus	3	1	4
Single ventricle heart	2	1	3
Other diagnosis^d^	4	2	6

"a" – Absent pulmonary valve, pulmonary atresia, right pulmonary artery stenosis, left pulmonary artery stenosis, pulmonary artery stenosis with bilateral aortopulmonary collaterals, multiple VSD, anomalus left anterior descending artery (LAD)

"b" – ASD and VSD, AVSD with hypoplastic arch, VSD with left ventricular outflow tract obstruction (LVOTO)

"c" – Coarctation of aorta, right ventricular outflow tract obstruction, ASD with bilateral superior vena cava

"d" – LVOTO, LVOTO with interrupted arch, pulmonary atresia with Ebstein anomaly, double outlet right ventricle with pulmonary atresia, anomalous LAD with myocardial infraction, pulmonary regurgitation with tricuspid anomaly

### Definitions

Low cardiac output is defined as persistent hypotension, oliguria (urine output < 0.5 ml/kg/hr) with clinical signs of tissue hypoperfusion despite maximum inotropic support. (Combination of dopamine, adrenaline, milrinone or enoximone and noradrenaline or vasopressin). In this study the term cardiorespiratory failure refers to severe respiratory failure leading to refractory hypoxemia resulting in cardiac dysfunction. Mechanical complications were complications related to ECMO circuit such as clot in the bladder or oxygenator failure.

Bleeding complications is defined as uncontrolled mediastinal bleeding despite correction of coagulopathy requiring surgical intervention. Abnormal neurology is defined as clinical signs of neurological dysfunction with definite radiological findings like cerebral bleeding or infarct on computerized tomographic scan of brain. Arrhythmias after ECMO support refer to the occurrence of non-sinus rhythm anytime after withdrawal of ECMO support. The term sepsis was defined as clinical evidence of sepsis with positive microbiology.

### ECMO circuit design

**Figure 1:**
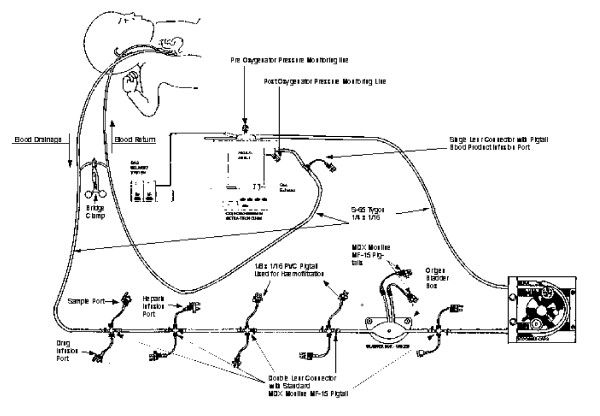
VA-ECMO circuit model.

The ECMO circuit consists of a venous cannula draining the blood by gravity to a compliant 60 ml silicon bladder, which is a servo regulating venous return monitor. A roller pump (Sorin Biomedical, New haven, West Sussex, UK) propels the blood from the bladder to an appropriately sized poly-methoxy pentane membrane oxygenator (Medos, Stolberg, Germany) with in-built heat exchanger (Betta-Tech controls, Milton Keynes, UK). Avecor silicone membrane oxygenators (Avecor Cardiovascular Products, Plymouth, Minnesota, USA) were used till 2000. The oxygenated blood from the oxygenator is returned to the patient through an arterial cannula. There is a connecting bridge between arterial and venous line of the ECMO circuit (Figure 1). All the patients in this study were supported by VA ECMO according to our published protocol [10].

### Patient management during ECMO support

#### Anticoagulation

Adequate anticoagulation was achieved by heparin sodium administration at a dose of 30–60 u/kg/hr to maintain an activated clotting time of 180 to 200 sec. Platelet count and fibrinogen were kept greater than 100,000/μl and 1 g/L respectively. Hemoglobin was maintained above 13 g/dl in all the patients. ACT and coagulation parameters were usually checked hourly and 8^th ^hourly respectively. From 2002 onwards we changed the ACT parameter to target between 160 to 180 seconds because of use of Actalyke Max ACT system [11].

#### Circulatory support

The primary goal of post cardiotomy ECMO support is to restore and maintain optimal tissue perfusion during myocardial recovery. ECMO flows were kept maximum, usually 150 ml/kg/min, as soon as the initiation of support and continued till satisfactory metabolic correction was obtained. Minimal inotropic support was continued during ECMO support (Dopamine 5–8 mcg/kg/min, Milrinone 0.25–0.50 mcg/kg/min) to facilitate left ventricular (LV) emptying against increased afterload and also helps to prevent overdistension of LV. Milrinone reduces the systemic vascular resistance thereby it attenuates the effect of increased afterload caused by ECMO support. One of the issues with ECMO for biventricular support is increasing LV end diastolic pressures and distension due to inadequate un-preloading of LV. Cardiac function was regularly assessed by echocardiograph (ECHO) with minimal ECMO flow. Atrial septostomy or left ventricular vent was used if there was evidence of LV distension. Patients were carefully assessed for adequacy of intracardiac repair by ECHO and where necessary by cardiac catheterization whilst on ECMO.

#### Ventilatory support

Once the condition of the patient was stabilized on ECMO, ventilatory settings were reduced to minimum. For the minimal setting, positive end-expiratory pressure was set at 5–10 cm H_2_O, peak inspiratory pressures were reduced to 20 cm H_2_O respiratory rate was set at 10/min and the inspired oxygen fraction was reduced to 40%. Since arterial oxygen content does not reflect the true picture of systemic hypoxia, mixed venous oxygen saturation was maintained between 70–85% during the ECMO support.

#### Fluid management

The patients were usually intravascularly volume overloaded so as to optimize of atrial filling pressure and maintain adequate cardiac output during the period of resuscitation. Once the ECMO was instituted, negative fluid balance was maintained in order to decrease the pulmonary intersitial edema. Diuretics were used to facilitate urine output of at least 2–3 ml/kg/hr. If the patient has persistent oliguria (Impending acute renal failure) despite diuretics, early institution of continuous venovenous hemofiltration (CVVH) along with ECMO support was employed.

#### Weaning

Full ECMO support was provided for minimum of 48 to 96 hours for almost all the patients. If the condition of patient improved to a satisfactory level, the flows were gradually reduced at hourly intervals over a 12 to 24 hour period. Once the patient is hemodynamically stable on minimal ECMO flow (30 ml/kg/min) with good recovery of myocardial contractility as evidenced by ECHO, patients were trailed off from ECMO support. If patient is successfully trailed off from ECMO, decannulation was done immediately and the cervical vessels were tied off during decannulation in all the patients.

### Statistical analysis

Univariate and multivariate analysis was performed using peri ECMO variables with Genstat Release version-6.1 (VSN International, Oxford, UK) statistical package. Variables included in the analysis are explained in Table 2. Analysis of the simple association, 2 × 2 table of association of death/survival classified by all the other binary factors, has been done using a chi-squared test and was checked by using Fisher exact test. Continuos variables were examined by logistic regression.

**Table 2:** Univariate analysis of peri ECMO variables

Variables	Survivors (N = 29)	Non survivors (N = 24)	p-Value
Age	589 days	452.5 days	NS
Male sex	15	14	NS
Weight	8.4 kg	7.2 kg	NS
Pre ECMO Metabolic acidosis	7.12 ± 0.07	7.08 ± 0.01	NS
Pre ECMO Arrhythmias	13	21	< 0.001
Cardiac arrest before cannulation	5	5	NS
Failure to wean from CPB	7	6	NS
Low cardiac output status	12	4	NS
Cardiorespiratory failure	5	9	NS
Bleeding complications	4	14	< 0.001
Use of RRT	10	20	< 0.001
Abnormal neurology	5	5	NS
Pulmonary complications	3	8	NS
Sepsis	3	6	NS
Mechanical complication of Circuit	10	15	NS
ECMO duration > 168 hrs	4	10	=0.024
Arrhythmias after ECMO support	9	22	=0.001
Cardiac arrest after ECMO support	6	16	< 0.001

CPB – Cardiopulmonary bypass; RRT – Renal replacement therapy.

## Results

Patient's age ranged from 1 to 3960 days (Median age: 150 days). Median weight of patient was 5.4 kg (range: 2.4 to 30 kg). Mean duration of ECMO support was 143 hours (range: 8 to 460 hours). Primary diagnosis in these patients were Tetralogy of Fallot's (TOF) with associated lesions in 16 patients, Transposition of Great Arteries (TGA) with associated lesions in 10 patients, VSD with associated lesions in 6 patients, Truncus arteriosus in 4 patients including one patient with DiGeorge syndrome, Univentricular heart in 3 patients [rudimentary right ventricle with VSD, double inlet left ventricle with pulmonary atresia, pulmonary atresia with intact ventricular septum], Total anomalous pulmonary venous drainage in 4 patients and AVSD in 3 patients. The remaining six patients had the following various lesions: Left ventricular outflow tract obstruction (LVOTO), LVOTO with interrupted arch, pulmonary atresia with Ebstein's anomaly, double outlet right ventricle with pulmonary atresia, anomalous left coronary artery with myocardial infraction and pulmonary regurgitation with tricuspid anomaly. All the associated lesions were described in Table 1 various operative procedures for these patients and survival were illustrated in Table 3.

**Table 3:** Operative procedures and survival

**Name of operation**	**Total number**	**Survival**
Repair of TOF +/- additional procedures	16 (9+7)	37%
Arterial switch +/- additional procedures	10 (7+3)	60%
VSD repair +/- additional procedures	6 (3+3)	67%
Complete AVSD repair +/- additional procedures	4 (2+2)	50%
Truncus arteriosus repair	4	75%
Repair of TAPVD	4	50%
Modified Fontan procedure	3	67%
Others^a^	6	67%

"a" – Repair of LVOT (Redo), Repair of LVOT with interrupted arch, Reimplant of left coronary (Takeuchi tunnel procedure), Left modified Blalock Taussig shunt, Repair of DORV and RVOT, Pulmonary and tricuspid valve repair

### Indications

The indications for ECMO support were a) low cardiac output (n = 16), b) failure to wean from cardiopulmonary bypass (n = 13), c) cardiac arrest in PICU (n = 10) and d) cardiorespiratory failure (n = 14).

### Overall outcome and survival based on indications

Thirty-five patients (66%) were successfully weaned off from ECMO support. Twenty-nine patients (54%) survived to hospital discharge. Twenty patients (37.7%) were alive after a mean follow up period of 75 months. Survivals to hospital discharge based on their indication for ECMO support were as follows: 75%(12/16) in low cardiac output patients, 54% (7/13) in failure to come off CPB patients, 50% (5/10) in cardiac arrest patients and 36% (5/14) in cardiorespiratory failure patients.

### Complications

Patient related complications such as bleeding and sepsis are charted in Table 4. The details of mechanical complications related to circuit are as follows: Thirteen patients (24%) had small blood clots identified in bladder box and bridging tube and 7 patients (13%) had clots in oxygenator. Three patients (6%)had oxygenator failure needed oxygenator changing and other two patients (4%)had ECMO circuit failure requiring new circuit. None of these mechanical complications were fatal.

**Table 4:** Complications during ECMO support

**Complications**	
Bleeding	36%
Neurological	19%
Sepsis^a^	17%
Renal	57%
Pulmonary^b^	21%
Mechanical^c^	47%

"a" – Chest infection (9.4%), Intraabdominal sepsis (3.7%), Sternal wound infection (1.8%), Cannulation site infection (1.8%).

"b" – Pneumothorax (11.3%), Pleural effusion (9.4%)

"c" – Mechanical complications of the ECMO circuit

### Mortality

Twenty-four patients (45%) died in hospital, eighteen patients (34%) died during ECMO support and six patients (11%) died in hospital after withdrawal of ECMO. About half of the patients died due to irreversible multiorgan failure (29%) and neurological damage (21%). Failed myocardial recovery (12%), hemorrhage (17%) including pulmonary and gastrointestinal hemorrhage and other causes (21%) including profound sepsis were responsible for the death of the remaining patients.

On univariate analysis the following factors were associated with increased hospital mortality: postoperative arrhythmias, duration of ECMO more than 168 hours, bleeding complications, requirement of renal replacement therapy (RRT) on ECMO, arrhythmias and cardiac arrest after ECMO support. On multivariate analysis, arrhythmias after ECMO, bleeding complications and abnormal neurology during ECMO were significantly associated with hospital mortality. Comparatively extra-thoracic cannulation had significantly less bleeding on ECMO (p = 0.031)

## Discussion

VA-ECMO is a partial cardiopulmonary support, which can be offered to any patient with potentially reversible cardiac, pulmonary or cardiopulmonary failure following cardiotomy. Recent studies have proved that it can be successfully used even after prolonged cardiopulmonary resuscitation [12,13]. Compared to adult cardiac support by VA-ECMO, the outcome of children who had VA-ECMO following the correction of congenital cardiac defect are very promising [3,9,14]. Interestingly there are no universally accepted standardized well-established criteria for indication and protocol to manage cardiac ECMO patients [15]. Since a wide range of complex congenital cardiac defects were supported by ECMO, there is considerable variation in the results published by different centres and therefore it complicates the issue of interpreting the published literature [15].

Achieving a successful outcome in this group of complicated cardiac patients is affected by many factors such as the timing of application, pre ECMO clinical status of patients and indications for ECMO support. Majority of published reports quotes that weaning from ECMO support as an outcome measures while some of them quotes as survival to hospital discharge [5,7,12,13,16]. So far only a few published reports have reported long-term as opposed to hospital survival[15,17,18]. In our study 66% of patients were successfully weaned off from ECMO and this compares well with the results of other experienced centres [3,7,9]. In this cohort, 54% of patients survived to hospital discharge, which is similar to other reports [7,16]. Among the hospital-discharged patients 69% are were alive and well for a mean follow up period of 75 months, this compares favorably with other published series[17,18]. In other words, 37.7% of patients were long-term survivors after post cardiotomy VA-ECMO support in this study. Most of the late deaths occurred a year after discharge in the community since there was no postmortem examination carried out in these patients the exact cause of deaths could not be identified. The pediatric cardiologists followed almost all the long-term survivors in the out patient clinic and they are doing well.

In this study survival to hospital discharge was better in patients who had ECMO support for low cardiac output compared to the other indications and all these patients had ECMO in the PICU because of failure of all conventional treatment modalities including induced hypothermia [19]. The duration of conventional treatment before these patients were supported on ECMO was raging between 2 to 48 hours (Mean 18.5 hours). In contrast to some of the published literature, in this series the institution of ECMO in the operating theatre for patients that had difficulties in coming off cardiopulmonary bypass had survival of 54% [5,20,21]. Most of the patients had complete surgical repair of their cardiac defects and in patients with shunt dependent pulmonary circulation the shunt was left open during ECMO support and this has been shown to be an important factor in achieving better survival in this group [22,23]. Even in the absence of a formal rapid deployment system (Extra-corporeal cardiopulmonary resuscitation program) the survival of patients supported by ECMO following cardiac arrest were 50% and this similar to other published reports [7,9,12,13]. Currently we are working to establish rapid deployment ECMO system in our unit. Based on the anatomical diagnosis, patient with TOF had comparatively poor outcome because majority of the patients in the non-survivor group had complicated associated lesions like distal pulmonary artery stenosis along with TOF (Table 1). Chaturvedi et al also observed poor survival among the TOF patients in their study with biventricular patients [24].

### Factors affecting the hospital survival identified by univariate analysis

Rhythm disturbances can often result in hemodynamic instability during the immediate postoperative period and it is a major cause of morbidity and mortality. Arrhythmias during the crucial period of myocardial recovery should always raise the possibility of progressive cardiac dysfunction or acute respiratory decompensation. Recurrent episodes of postoperative arrhythmias before the initiation of ECMO were significantly associated with the hospital mortality in this study (p < 0.001). Most of the arrhythmias were resistant to anti-arrhythmatic medications. Hoskote, A and collegues also found that refractory tachydysrhythmia before initiation of ECMO support was a risk factor for mortality in functional single ventricle patients[25]. Extracorporeal life support has also been used successfully for the treatment of supraventricular tachycardia (SVT) as well [26].

Some of these patients required longer duration of mechanical support to get adequate myocardial recovery. Severe initial myocardial insult before the initiation of ECMO support could be the likely reason for prolonged myocardial recovery time. In this study it was found that one fourth of patients (26.4%) required ECMO support over a week and four patients (7.54%) needed more than two weeks. Statistically it was observed that duration of ECMO more than 168 hours is clearly associated with hospital mortality (p < 0.001). Previous studies have demonstrated the fact that if the patient needed a longer duration of ECMO support, their survival tends to be poor[3,7,22,24]. In fact, Duncan et al observed poorer outcomes even in the children who were supported by VAD for more than 48 hours[7].

More than half of patients (57%) required CVVH in this study, the need of RRT on ECLS in these patients implies the severity of secondary organ dysfunction. The majority of these patients were treated by continuous venovenous hemofiltration while few of them had peritoneal dialysis. In this cohort, requirement of renal replacement therapy during ECMO support was identified as a significant risk factor for hospital mortality (p < 0.001). Kolovos et al also demonstrated that the post cardiotomy ECMO supported children requiring hemofiltration were five times more likely die than other patients [9].

### Factors affecting the hospital survival identified by multivariate analysis

The incidence of neurological complications during VA-ECMO support varies from 22% to 31% [7,16,17]. In our study total of ten patients (21%) had abnormal neurology; three of them had cardiopulmonary resuscitation (CPR) for more than 45 minutes before initiation of ECMO. It is difficult to attribute ECMO support was the reason for neurological complications in these 3 patients. Kulik et al mentioned about thirty percent of patients with neurological complication who had previous CPR before ECMO, moreover neurological complications was one of the reasons for withdrawal of ECMO support in their study [27]. In our study Four patients had intracranial bleeding (8%), Two patients had localized cerebral infracts (4%), Three patients had hypoxic ischemic encephalopathy (5%) and one patient had cerebral atrophy (2%). we found that abnormal neurology is one of the variables strongly associated with hospital mortality (p < 0.001). In this cohort only two of long-term survivors had functional neurological deficit (Hemiperesis) where as 39% of long-term survivors had neurological deficits in Chow et al study [17]. They also found that CPR prior to ECLS was a predictor of hospital death but whether there is a relation between the CPR prior to ECLS and neurological deficit is not clear in their study [17]. Where as Chaturvedi and colleagues have proved that brain death was very strongly associated with CPR before initiation of ECMO [24].

Bleeding complications are an inevitable problem during this life saving therapy. Heparin is not the only factor responsible for bleeding in these patients since most of them were bound to have altered homeostasis resulting in deranged hemostasis. Mediastinal bleeding requiring surgical reexploration was another risk factor significantly affecting hospital survival in this group of patients (p < 0.001). In this study 36% of patients had intrathoracic bleeding within 48 hours of initiation of ECMO. Once the abnormal coagulation was corrected with Fresh frozen plasma, Cryoprecipitate and platelet transfusion. Heparin was withheld temporarily in these patients to control the bleeding. Aprotinin and AMICAR were also used. Failure of these conservative measures resulted surgical reexploration. Upon reopening the chest almost more than 90% of patient no surgical source of bleeding was found. Their sternum left open with skin closed for 24 to 48 hours. Majority of them had more than one surgical reexploration. Meliones JN and collegues were also identified mediastinal bleeding as risk factor for mortality in their review of extracorporeal life support organization registry [28]. The initiation of ECMO leads to an initial worsening of coagulopathy in these sick patients[29] Whether using heparin coated extra-corporeal circuit [30] or using recombinant Factor VIIa would make any difference in these patients needs further evaluation[31]. In fact, Ahoran et al reported all the patients who had transthoracic ECMO cannulation needed surgical reexploration for bleeding but the actual incidence surgical bleeding in that group was not mentioned [3]. The relationship between type of cannulation and bleeding complications in our study will be discussed below.

Rhythm disturbances after withdrawal of ECMO are potentially serious complications adversely affecting the outcome. Occurrence of arrhythmias in these patients may be due to electrolyte abnormalities, metabolic disturbances (acidosis) and poor myocardial recovery despite maximal ECMO support. Nine patients (29%) developed recurrent arrhythmias after withdrawal of ECMO. Six patients had SVT (Junctional ectopic tachycardia in five, atrial flutter in one patient) and three patients had ventricular tachycardia. These patients were treated with appropriate anti-arrhythmatic medications and electro-cardioversion. In this study arrhythmias after withdrawal of ECMO support was identified as a risk factor for hospital mortality (p < 0.001). Becker et al found that cardiovascular complications including arrhythmias have significant negative impact on the survival of the ECMO patients [32]. Cardiac complications were also noted to be one of the major causes for mortality in the ELSO registry data [28]. Identifying these high-risk patients with poor myocardial recovery and address them with bridge to transplant or recovery might improve the survival [33].

### Cannulation technique

Appropriate size of the cannula is the crucial factor in providing the support by extracorporeal circuit. In these patients even if cannulation were done in the operating theatre, the preferred type of cannulation was cervical. The cannulation technique has been described previously [34]. Seventy nine percent of patients were cannulated via the internal jugular vein and carotid artery while only twenty one percent had transthoracic cannulation. In this study we identified that the extrathoracic cannulation was associated with significantly less bleeding complications (p = 0.031). Duncan and colleagues also found this fact in their study involving both surgical and non-surgical patients [7]. Moreover the relationship between cannulation type and the abnormal neurology was assessed using Fisher's exact test and was not found to be significant (p = 0.67).

### Study limitations

This is a retrospective study from a single centre. Sample size is relatively small and only three patients with univentricular hearts were involved. Post cardiotomy paediatric patients following surgical correction of congenital cardiac defect who required VA-ECMO only were included in this study. Some laboratory variables like lactate could not be included in the analysis because of incomplete data. In spite of the limitations, our study is one of the few reported a long-term survival of pediatric patients following VA-ECMO support.

## Conclusion

Our long-term data confirms that Veno-arterial ECMO can provide an effective mechanical circulatory support following adequate surgical repair of CCD in children. Extra thoracic cannulation is associated with less bleeding complications during ECMO support. Abnormal neurology, bleeding complications on ECMO and arrhythmias after ECMO support, are poor prognostic indicators for hospital survival.

## Abbreviations

AVSD: Atrioventricular septal defect

ACT: Activated clotting time

CCD: congenital cardiac defects

CPB: cardiopulmonary bypass

CPR: cardiopulmonary resuscitation

CVVH: continuos venovenous hemofiltration

ECHO: Echocardiography

IABP: Intra aortic balloon pump

LVOTO: Left ventricular outflow tract obstruction

PICU: pediatric intensive care units

RRT: renal replacement therapy

VAD: Ventricular assist devices

VA-ECMO: Venoarterial Extracorporeal membrane oxygenation

TGA: Transposition of great arteries

## Authors' contributions

**SKB**: have made substantial contributions to conception and design, acquisition of data, analysis, interpretation of data and drafting the manuscript

**RT**: have been involved in drafting the manuscript

**MA**: Data collection

**KKA**: Data collection and analysis

**GJP**: have been involved in revising it critically for important intellectual content.

**AWS**: have been involved in approving final version for submission.

**RKF**: have been involved in revising it critically for important intellectual content.
